# Dr. William H. Maynard

**Published:** 1916-03

**Authors:** 


**Dr. William H. Maynard,** Syracuse, 1883, died at Syracuse Jan. 15, aged 57.

